# Wild foods as drivers of blood ergothioneine and selenoneine concentrations among Inuit living in Nunavik: results from the cross-sectional *Qanuilirpitaa?* 2017 survey

**DOI:** 10.1016/j.ajcnut.2025.05.009

**Published:** 2025-07-04

**Authors:** Pierre Ayotte, Mélanie Lemire, Pierre Dumas, Adel Achouba, Marcos Yedjenou, Ariane B Barrette, Nathalie Ouellet, Matthew Little, Amira Aker

**Affiliations:** 1 Département de médecine sociale et préventive, Université Laval, Québec, QC, Canada; 2 Axe santé des populations et pratiques optimales en santé, Centre de recherche du CHU de Québec-Université Laval, Québec, QC, Canada; 3 Centre de toxicologie du Québec, Institut national de santé publique du Québec (INSPQ), Québec, QC, Canada; 4 Institut de biologie intégrative et des systèmes, Université Laval, Québec, QC, Canada; 5 Département de biologie, Université Laval, Québec, QC, Canada; 6 School of Public Health and Social Policy, University of Victoria, Victoria, BC, Canada

**Keywords:** ergothioneine, selenoneine, blood levels, dietary profiles, Inuit of Nunavik

## Abstract

**Background:**

Wild foods traditionally harvested by Inuit, also called country foods, are potential sources of ergothioneine and selenoneine, 2 closely related antioxidants with potential health benefits.

**Objective:**

To determine concentrations of these compounds and methylated metabolites in blood samples from 1291 Nunavik residents (Nunavimmiut) aged ≥16 y who participated in the cross-sectional *Qanuilirpitaa?* 2017 Health Survey and associated dietary habits.

**Methods:**

Blood levels were measured using isotope dilution-liquid chromatography-tandem MS. Associations with dietary profiles or selected dietary habits (documented by a food frequency questionnaire) were investigated using multivariate models.

**Results:**

Geometric mean concentrations (95% confidence interval [CI]) of ergothioneine, S-methyl-ergothioneine, selenoneine, and Se-methyl-selenoneine, were 92.5 mg/L (88.4, 96.8), 139 μg/L (133, 146), 355 μg/L (328, 385) and 11.6 μg/L (10.7, 12.5), respectively. Geometric mean ratios (GMR) (95% CI) comparing females with males were 1.27 (1.18, 1.39) and 1.82 (1.57, 2.11) for ergothioneine and selenoneine, respectively. GMR comparing ≥60 y olds to youth aged 16 to 19 y were 1.75 (1.52, 2.02) and 2.78 (2.04, 3.69) for ergothioneine and selenoneine, respectively. Blood selenoneine concentrations of Hudson Strait residents exceeded those of Ungava Bay (2.38 [1.97, 2.86]) and Hudson Bay residents (2.70 [2.22, 3.28]). GMR comparing the high-country food consumption profile with none (or very low) profile were 1.33 (1.10, 1.61) and 2.35 (1.65, 3.36) for ergothioneine and selenoneine, respectively. Country foods positively associated with ergothioneine concentrations included Arctic char (1.07 [1.04, 1.10]) and caribou meat (1.06 [1.03, 1.10]), whereas country foods positively linked to selenoneine concentrations comprised Arctic char (1.07 [1.02, 1.12]) and beluga mattaaq (1.15 [1.08, 1.22]).

**Conclusions:**

Although comparative data are limited, blood selenoneine and ergothioneine concentrations among Nunavimmiut appear substantially higher than in other non-Indigenous populations. Access to country food is important to maintain the dietary intake of these bioactive food components that may be beneficial for the health of Nunavimmiut.

## Introduction

**FIGURE 1:**
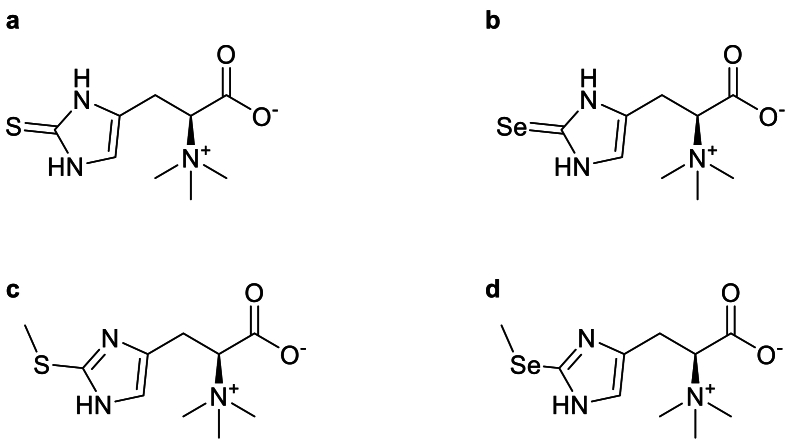
Chemical structure of ergothioneine (a), selenoneine (b), S-methyl-ergothioneine (c) and Se-methyl-selenoneine (d)

Ergothioneine and selenoneine are naturally occurring histidine betaine derivatives exhibiting strong antioxidant activity and several potential health benefits [1,2]. Ergothioneine (Figure 1A), first isolated in 1906 by Tanret from *Claviceps purpurea* [3], is widely distributed in plants, fungi, and animals [4]. Selenoneine, the selenium isologue of ergothioneine (Figure 1B), was first identified in the blood of bluefin tuna by Yamashita and Yamashita in 2010 [5] and subsequently in other marine organisms [[6], [7], [8]]. These compounds are not synthesized by animals but can be acquired from food and distributed to body organs and tissues via the ergothioneine transporter (ETT), coded by the human gene *SLC22A4* (solute carrier family 22 member 4) [9]. In the blood, both compounds accumulate in red blood cells (RBCs) in concentrations much higher than in the plasma [6,10,11]. Ergothioneine was also shown to accumulate in multiple tissues of mice, including liver, kidney, and brain [11]. Methylation appears to be a major biotransformation pathway of ergothioneine [10] and selenoneine [12], leading to the formation of S-methyl-ergothioneine (Figure 1C) and Se-methyl-selenoneine, respectively (Figure 1D). Despite mounting interest in these compounds, data on blood levels in human populations are limited.

**FIGURE 2:**
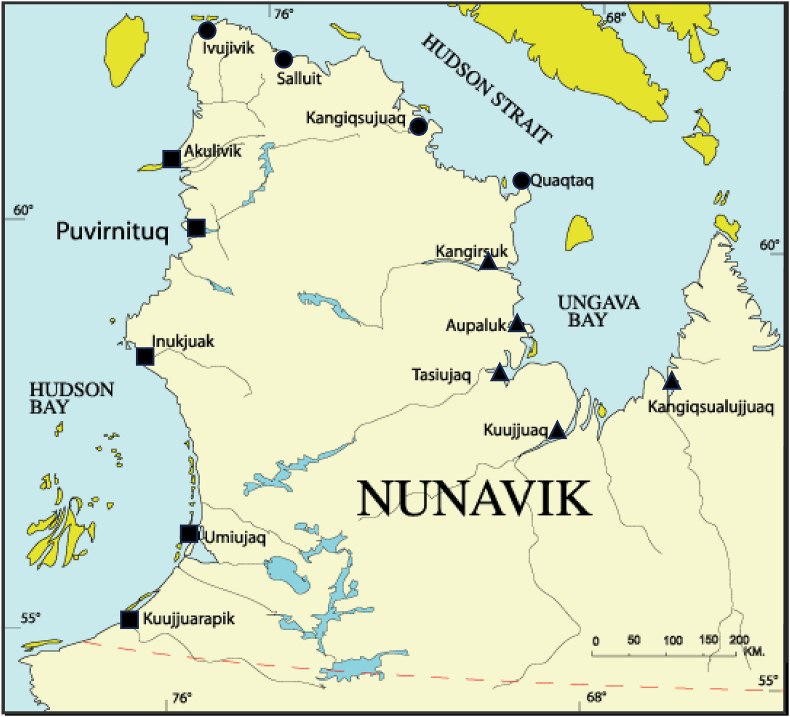
Map of Nunavik (source: Makivvik Corporation) ( ■ ) Hudson Bay communities; ( • ) Hudson Strait communities; ( ▲ ) Ungava Bay communities

Nunavik, a vast territory located north of the 55^th^ parallel in northern Québec (Figure 2), Canada, is home to 12,000 Inuit [13]. Inuit living in Nunavik (Nunavimmiut in Inuktitut) have subsisted for thousands of years on traditional “country” foods harvested from the local environment, including land and marine mammals, fish, bivalves, migratory birds, and foraged foods. Although market foods constitute a major part of Nunavimmiut diets nowadays, country foods such as berries, caribou meat, Arctic char, and beluga mattaaq (skin with the underlying fat layer) are still highly praised and frequently consumed [14]. We previously reported that selenoneine was a major selenium species in beluga skin and RBCs of Nunavimmiut [6,15]. Furthermore, preliminary results from our laboratory indicating that ergothioneine is present in several country foods popular in Nunavik (Supplemental Table 1); we hypothesized that the diet of Nunavimmiut would result in high blood concentrations of ergothioneine as well.

Using our newly developed isotope dilution-liquid chromatography-tandem MS (ID-LC-MS/MS) method [16], we measured concentrations of ergothioneine, selenoneine, and their methylated metabolites in blood samples of Nunavimmiut aged ≥16 who participated in the *Qanuilirpitaa?* 2017 Nunavik Health Survey. Concentrations are reported according to sex, age group, and ecological subregion of residence (Hudson Bay, Hudson Strait, Ungava Bay), among which country foods harvested and consumed differ [14,17]. We also examined associations between blood concentrations of these compounds and dietary profiles [18] and consumption frequencies of selected country and market foods.

## Methods

### Population

The *Qanuilirpitaa?* 2017 Nunavik Inuit Health Survey is a population health survey conducted for and by Inuit across the 14 villages of Nunavik, Canada. A total of 1326 respondents were surveyed between August 19 and October 5, 2017. The survey aimed at recruiting a representative sample to assess the health status of Nunavimmiut. Toward this end, a stratified proportional model based on age group (16–29, 30–49, and ≥50 y) and region (Hudson Bay, Hudson Strait, and Ungava Bay; [Figure 2]) was implemented for respondent selection, targeting permanent residents aged ≥16 y. People living in institutional settings and those with active tuberculosis were excluded. All survey and clinical tests were performed on board the Amundsen, a Canadian Coast Guard Icebreaker. Survey questionnaires were administered by interviewers in Inuktitut, English, or French. The overall participation rate was relatively low at 36.7%, mainly due to the high noncontact rate because of people being outside their homes (i.e., hunting, fishing, gathering) or away from their community at the time of the ship’s arrival. Of those who were contacted, 79.7% agreed to participate. Additional details on the methodology and the study population are provided elsewhere [19,20]. The final sample of 1291 Nunavimmiut for the present study was obtained after excluding 33 pregnant females and 2 participants without a blood sample. Additional participants who did not complete the dietary questionnaire were excluded when testing associations between dietary habits and blood concentrations of the dietary antioxidants (*N* = 1143).

Ethical approval was received from the Comité d’éthique de la recherche du Centre Hospitalier Universitaire de Québec - Université Laval (no. 2016–2499). The survey was conducted in close collaboration with major Nunavik organizations (Nunavik Regional Board of Health and Social Services and the health centers, the Kativik Regional Government, Makivvik Corporation, Kativik Ilisarniliriniq, Avataq Cultural Institute, and Qarjuit Youth Council) and guided by the OCAP principles (Ownership, Control, Access, and Possession). The Nunavik Health Surveys Committee (NHSC) evaluates the relevance of research questions for the region and approves data and biological sample requests. This committee brings together representatives from Nunavik partner organizations, the INSPQ, and academic researchers. The NHSC also met with the researchers to discuss results and provide cointerpretation of the data, taking into consideration Inuit culture and Nunavik perspective. Comments provided by committee members were considered in preparing the final version of the manuscript, which was approved by the NHSC.

### Blood sampling, storage, and analysis

As part of the survey, blood samples were obtained from participants and analyzed for various nutritional biomarkers of and biomarkers of exposure to environmental contaminants. Trained nurses collected blood samples via cubital venipuncture in vacutainers containing K_2_-EDTA as the anticoagulant. Aliquots of whole blood samples were transferred into polypropylene tubes and stored frozen at −80^o^C for subsequent analyses.

Concentrations of ergothioneine, selenoneine, and their methylated metabolites were determined in whole blood samples using our newly developed and validated ID-LC-MS/MS method [16]. Briefly, a 50-μL aliquot of blood was spiked with isotopically labeled standards (ergothioneine-d_9_, S-methyl-ergothioneine-d_9_, ^77^Se-selenoneine, ^77^Se-methyl-selenoneine) and mixed with 200 μL of a 50-mM dithiothreitol aqueous solution. The resulting mixture was then filtered through a 3-kDa cutoff 0.5-mL centrifugal filter (EMD Millipore, Omaha, NB) at 12,000 × g for 45 min at room temperature. A 20-μL aliquot of the filtrate was mixed with 180 μL of mobile phase A (see below), and the resulting solution was analyzed by ID-LC–MS/MS.

Separation of analytes was performed on an Acquity I Class ultraperformance liquid chromatography system (UPLC) (Waters) equipped with an Acquity HSS T3, 150 × 2.1 mm, 1.8 μm column (Waters) and operated at 30°C with a flow rate of 0.4 mL/min. Mobile phases consisted of (A) an aqueous solution containing 0.1% formic acid and 10 mM ammonium formate and (B) acetonitrile. The gradient elution program was as follows: 0 to 1.6 min 100% (A), 1.6–2 min gradient to 10% (A), 2 to 3.6 min at 10% (A), and 3.6 to 8 min back to 100% (A). The UPLC system was coupled to a Xevo TQS micro triple quadrupole mass spectrometer (Waters) equipped with an electrospray ionization source that operated in positive mode. Argon was used as the collision gas, and acquisition was carried out using multiple reaction monitoring. Matrix matched calibration curves were prepared at the following concentrations: 0, 5, 12.5, 50, 125, 250, 500 mg/L for ergothioneine; 0, 5, 12.5, 50, 125, 250, 500 μg/L for S-methyl-ergothioneine; 0, 6, 12, 30, 120, 300, 600, 2400, 4800 μg/L for selenoneine; 0, 2, 4, 10, 40, 100, 200, 400, 800 μg/L for Se-methyl-selenoneine. We prepared quality controls (QC)—low (L), medium (M), and high (H)—using pooled blood samples that were assembled based on the previously determined total selenium concentration of blood samples from the survey participants [21]. Limits of detection for ergothioneine, S-methyl-ergothioneine, selenoneine, and Se-methyl-selenoneine were 0.75 mg/L, 0.76 μg/L, 2.7 μg/L, and 0.5 μg/L, respectively. The interday precision values (% CV), evaluated using the endogenous concentrations of QC samples, were all inferior to 10%. Additional information on the validation and performance of this method can be found elsewhere [16].

### Food frequency consumption and dietary profiles

Study participants were asked to complete a food frequency questionnaire (FFQ) to measure the consumption frequency of a range of market and country foods in the previous 3 mo. The consumption of each food item was rated on a scale of 1 (never consumed or less than once a month) to 7 (consumed ≥4 times a day).

We used 2 types of dietary profiles previously created by Aker et al. [18] using latent profile analysis. The global food consumption profiles comprised all food items, including both market and country food items, and were the following: *1*) market food dominant, *2*) country food dominant, *3*) diverse consumption, and *4*) low consumption. The second group of dietary profiles included only country food items and were as follows: *1*) no (or very low) consumption; *2*) low consumption; *3*) moderate consumption; and *4*) high consumption. A description of the main foods and consumption frequencies among the different consumption profiles can be found in Aker et al. [18]. Country food items considered as potential sources of ergothioneine and/or selenoneine in models were selected according to published and unpublished data from our laboratory (Supplemental Table 1). Fifteen country foods were considered in ergothioneine models: dried beluga meat; beluga meat (fresh, cooked or frozen); beluga mattaaq; seal meat; seal liver; dried caribou meat; caribou meat (fresh, cooked or frozen); ptarmigan or partridge; snow or Canada goose; dried fish; lake trout; Arctic char; other fish; mollusks or urchins; and seaweed. The same country foods were considered in selenoneine models, except for caribou meat, which does not contain any selenoneine. Market foods considered as potential sources of ergothioneine were selected based on data compiled by Tian et al. [4]. The following market foods were included: red and processed meat (a summation variable of 6 dietary items in the FFQ: processed meat; sausages; hot dogs; bacon; beef jerky; hamburger, beef or pork as main dish); chicken or turkey (a summation variable of 2 dietary items in the FFQ: chicken or turkey parts; chicken nuggets or chicken wings or fried chicken); chicken eggs; beans or lentils or chickpeas; nuts or peanuts or sunflower seeds; other vegetables (peppers, onions, corn, cucumber, celery, or mixed vegetables) or mushrooms; hot cereals (oatmeal). Because tuna was recently shown to contain both ergothioneine and selenoneine [22], canned fish (tuna, salmon, or sardines) was considered a potential source of both antioxidant compounds. The correspondence between FFQ consumption categories and monthly frequencies used in models is presented in Supplemental Table 2.

### Covariates

Anthropometric measurements were collected by nurses or trained interviewers. Height was measured with a tape measure graduated in centimeters (cm) while the participant was standing on a hard surface without shoes. Body weight was measured on an InBody scale (InBody 570, InBody Canada). Waist circumference (WC) was measured at the end of a normal expiration with the tape measure positioned at the midpoint between the last floating rib and the top of the iliac crest, and the mean of 2 measurements was used [19]. A third measurement was performed if the difference between the first 2 values was > 1 cm and was used as the final value.

BMI and WC were considered as obesity indicators in adjusted models. BMI was calculated by dividing the weight in kilograms by the square of the height in meters (kg/m^2^) and was classified according to the scheme elaborated by the National Institutes of Health [23]. Participants with a BMI value < 18.5 kg/m^2^ were classified as underweight, ≥ 18.5 to 24.9 kg/m^2^ normal weight, ≥ 25 kg/m^2^ overweight, ≥ 30 kg/m^2^ class 1 obese, ≥ 35 kg/m^2^ class 2 obese, and ≥ 39 kg/m^2^ class 3 obese. The underweight and the normal BMI categories were combined because the underweight category represented 2.3% of the population sample.

Blood hemoglobin concentration was included in the models because selenoneine reportedly binds to hemoglobin [5], and both selenoneine and ergothioneine are concentrated in RBCs [6,9]. Whole blood hemoglobin analysis was performed immediately upon collection using the portable DxH500 hematology analyzer from Beckman-Coulter.

We also considered smoking as a potential confounder in adjusted models. The current smoking status was determined from the question: “At the present time, do you smoke cigarettes every day, occasionally, or not at all?”

### Statistical analyses

Sample characteristics were described using proportions and medians. Concentrations of ergothioneine, selenoneine, and their metabolites were compared between sexes and among age groups and ecological subregions using unadjusted linear regression models. Intercorrelations between these analytes were also calculated. Adjusted linear regression models were used to compare concentrations of compounds according to food consumption profiles (global or country food specific) identified by Aker et al. [18], and according to consumption frequencies of selected country and market foods. Sex, age, current smoking status, WC (or BMI), and blood hemoglobin concentration were included in models as adjustment variables (all but sex and smoking as continuous variables). Food items were analyzed separately in covariate-adjusted univariate models and jointly in covariate-adjusted multivariate models. A first multivariate model including all food items was reduced to a final model containing only statistically significant food items by removing nonsignificant items one by one. Log-transformed concentrations of ergothioneine, selenoneine, and their methylated metabolites were used in all analyses. Therefore, results are presented as geometric means (GM) and geometric mean ratios (GMR). For food consumption frequency variables, GMR corresponds to the doubling of monthly consumption as these variables were base 2 log-transformed.

Statistical analyses were performed using the SAS 9.4 software (SAS Institute Inc.). All analyses accounted for the complex sampling strategy of the survey using SAS 9.4 “survey” procedures: estimates were computed using original sample weights, and variances of estimates were calculated using the bootstrap (resampling with replacement) method with 500 sets of bootstrap weights. Weighted estimates are presented, and N values are unweighted. A bilateral alpha level of 5% was used for statistical significance.

## Results

### Characteristics of participants

Selected characteristics of the population sample (*N* = 1291) are listed in Table 1. Nearly twice as many females as males participated in the survey (840 females and 451 males). Forty-one percent of participants were in the 16-29 y age group, and 59% were in the ≥30 y category. Hudson Bay was the most represented ecological region, with 42% of participants living in this region. Seventy-one percent of respondents were daily smokers.

**TABLE 1:** Characteristics of Nunavimmiut aged 16 y and over by sex, *Qanuilirpitaa*? 2017 Health Survey 1.

			Total			Females			Males		
			(*N* = 1291)			(*N* = 840)			(*N* = 451)		
			N	% or median	SE	N	% or median	SE	N	% or median	SE
Sociodemographic characteristics											
	Age categories (y)										
		16-19	222	15.2	0.96	135	12.3	0.97	87	17.9	1.60
		20-29	297	25.9	1.01	210	27.5	1.09	87	24.4	1.65
		30-39	199	17.6	0.97	132	17.7	1.19	67	17.5	1.59
		40-49	202	16.8	1.03	130	16.9	1.18	72	16.7	1.74
		50-59	211	13.7	0.80	144	15.8	0.98	67	11.6	1.20
		60+	160	10.9	0.83	89	9.9	0.91	71	11.9	1.29
	Ecological subregions										
		Hudson Bay	458	41.6	0.18	280	40.8	0.36	178	42.5	0.09
		Hudson Strait	296	24.2	0.14	200	24.7	0.27	96	23.8	0.11
		Ungava Bay	537	34.1	0.14	360	34.5	0.26	177	33.7	0.11
Obesity indicators											
	Body mass index (kg/m^2^)										
		Underweight + Normal weight (< 25)	504	44.6	1.63	279	36.9	1.82	225	51.2	2.62
		Overweight (≥ 25)	312	25.5	1.43	202	27.6	1.68	110	23.7	2.24
		Type 1 obesity (≥ 30)	226	18.6	1.22	156	21.3	1.50	70	16.3	1.85
		Type 2 obesity (≥ 35)	79	6.3	0.76	55	7.8	1.01	24	5.1	1.08
		Type 3 obesity (≥ 39)	61	4.9	0.72	46	6.4	0.93	15	3.7	1.02
	Waist circumference (cm; median, SE)		—	91.0	0.86	—	93.7	0.68	—	86.8	1.21
Anemia biomarker											
	Blood hemoglobin concentration (g/L; median, SE)		—	137	0.45	—	130	0.50	—	144	0.67
Lifestyle habits											
	Current smoking status										
		Daily	879	71.1	1.39	589	73.3	1.62	290	69.2	2.18
		Occasionally	111	8.2	0.90	70	7.7	0.92	41	8.6	1.45
		Not at all	282	20.7	1.25	169	19.1	1.39	113	22.2	1.95
	Global food consumption profiles^2^										
		Market food dominant	484	41.1	1.71	329	44.1	1.99	155	38.2	2.68
		Country food dominant	114	12.7	1.18	46	6.70	1.21	68	18.3	2.06
		Diverse	279	23.6	1.44	187	25.4	1.76	92	22.0	2.23
		Low	266	22.6	1.26	179	23.8	1.62	87	21.5	2.05
	Country food consumption profiles^2^										
		None (or very low)	357	30.6	1.45	242	32.7	1.76	115	28.6	2.25
		Low	513	42.6	1.57	354	46.7	1.95	159	38.8	2.46
		Moderate	172	15.6	1.19	107	15.6	1.52	65	15.6	1.87
		High	101	11.2	1.13	38	5.0	0.92	63	17.0	2.03
	Food frequency consumption^2^ (times/mo; median, SE)										
	Country foods										
		Arctic char	—	1.66	0.02	—	1.55	0.02	—	1.81	0.19
		Other fish	—	0.50	0.10	—	0.50	0.13	—	0.50	0.14
		Dried fish	—	1.35	0.02	—	1.24	0.02	—	1.45	0.04
		Lake_trout	—	0.55	0.03	—	0.50	0.05	—	0.70	0.04
		Beluga meat (fresh, cooked, or frozen)	—	0.50	0.07	—	0.50	0.10	—	0.50	0.07
		Dried beluga meat	—	0.63	0.03	—	0.50	0.04	—	0.77	0.04
		Beluga mattaaq	—	1.14	0.02	—	1.10	0.02	—	1.19	0.03
		Seal meat	—	0.50	0.04	—	0.50	0.05	—	0.54	0.05
		Seal liver	—	0.50	0.07	—	0.50	0.10	—	0.50	0.10
		Caribou meat (fresh, cooked, or frozen)	—	2.15	0.36	—	2.20	0.32	—	2.10	0.50
		Dried caribou meat	—	1.86	0.06	—	1.71	0.03	—	2.10	0.48
		Ptarmigan or partridge	—	0.50	0.05	—	0.50	0.06	—	0.50	0.07
		Snow or Canada goose	—	1.14	0.02	—	0.98	0.02	—	1.29	0.03
		Mollusks or urchins	—	0.81	0.02	—	0.77	0.02	—	0.85	0.03
		Seaweed	—	0.50	0.09	—	0.50	0.08	—	0.50	0.19
	Market foods										
		Chicken eggs	—	7.65	0.43	—	6.97	0.52	—	8.25	0.63
		Chicken or turkey	—	6.09	0.23	—	5.68	0.23	—	6.48	0.31
		Red and processed meat	—	26.8	1.05	—	23.4	1.08	—	30.6	2.42
		Canned fish	—	0.50	0.07	—	0.50	0.11	—	0.50	0.08
		Beans or lentils or chickpeas	—	0.50	0.14	—	0.50	0.20	—	0.50	0.17
		Nuts or peanuts or sunflower seeds	—	0.51	0.05	—	0.63	0.05	—	0.50	0.08
		Other vegetables or mushrooms	—	3.19	0.11	—	3.67	0.25	—	2.81	0.16
		Hot cereals (oatmeal)	—	0.50	0.10	—	0.50	0.10	—	0.50	1.19

1 Values are N, % and SE for categorical variables, median and SE for continuous variables.

2 Food frequency consumption of selected foods and food consumption profiles derived from the food frequency questionnaire data (total *n* = 1143; females, *n* = 741; males, *n* = 402).

Slightly more than half of males (51%) were in the underweight/normal weight category, compared with 37% of females, whereas nearly 36% of females were in the obese category (types 1–3), compared with 25% of males. Median WC was 6.9 cm greater in females than in males. Median hemoglobin concentration in males exceeded that of females by 14 g/L.

Information on diet was available for 1143 participants (741 females and 402 males). Among global dietary profiles, which consider both market and country foods, the market food dominant category had the greatest proportion of participants (41.1%). Proportionally, more males than females had a country food dominant consumption profile (18.3% compared with 6.7%). When considering only country food consumption profiles, about one-third of participants consumed very little or did not consume country food at all. Proportionally, more males than females were in the high-country food consumption profile (17.0% compared with 5.0%). Among country foods considered as potential dietary sources of either ergothioneine or selenoneine, caribou meat (fresh, cooked, or frozen) and dried caribou meat were each consumed approximately twice a month, and beluga mattaaq once a month (median consumption frequencies).

### Blood concentrations of ergothioneine, selenoneine, and methylated metabolites

Ergothioneine, S-methyl-ergothioneine, and selenoneine were detected in 100% of blood samples, and Se-methyl-selenoneine in 95% of them (Table 2). On a molar basis, GM concentration of ergothioneine (92.5 mg/L, corresponding to 400 μmol/L) was in excess of 300-fold greater than that of its selenium isologue selenoneine (0.36 mg/L, corresponding to 1.30 μmol/L). On average, selenoneine represented about one-third of the total selenium concentration in whole blood (GM = 34%, 95% CI: = 32, 36) but constituted 82% (80 – 84) of the total selenium concentration in the 5% of individuals at the higher end of the blood selenium distribution (Supplemental Table 3).

**TABLE 2:** Blood concentrations of ergothioneine, selenoneine, and methylated metabolites in Nunavimmmiut aged ≥16 y, *Qanuilirpitaa*? 2017 Health Survey^1^.

	Total (*N* = 1291)		Females (*N* = 840)		Males (*N* = 451)	
Blood metabolite	GM	95% CI	GM	95% CI	GM	95% CI
Ergothioneine (mg/L)	92.5	88.4, 96.8	105	100, 110	82.1	76.2, 88.4
S-methyl-ergothioneine (μg/L)	139	133, 146	154	148, 161	126	117, 136
Selenoneine (μg/L)	355	328, 385	483	448, 521	265	232, 303
Se-methyl-selenoneine (μg/L)	11.6	10.7, 12.5	15.4	14.3, 16.6	8.80	7.70, 10.1

Abbreviations: GM, geometric mean.

1 Unadjusted geometric means and 95% confidence intervals are presented. % detected = 100% for ergothioneine, S-methyl-ergothioneine and selenoneine; 95% for Se-methyl-selenoneine. All geometric mean ratios (females/males) are statistically significant (*P* < 0.001) according to the *t* values for the difference of means obtained from unadjusted weighted linear regression models with a bootstrap variance.

Blood concentrations of ergothioneine and selenoneine were significantly higher in females than in males (Table 2). GMR (95% CI) comparing females to males were 1.27 (1.18, 1.39) and 1.82 (1.57, 2.11) for ergothioneine and selenoneine, respectively.

**FIGURE 3:**
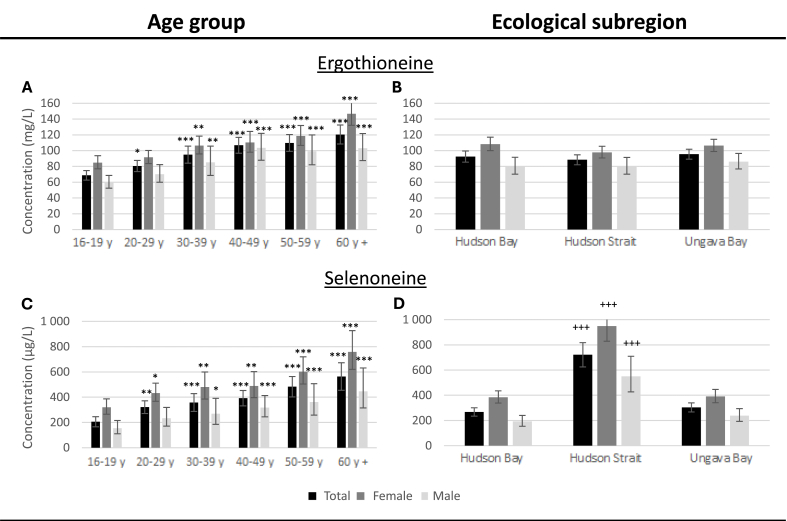
Unadjusted geometric mean blood concentrations of ergothioneine, selenoneine, and methylated metabolites (+/- 95% confidence interval) in Nunavimmiut aged 16 and over by age group and ecological subregion, *Qanuilirpitaa*? 2017 Health Survey. Statistical significance was tested using *t* values for the difference of means obtained from unadjusted linear regression models with indicator variables for age groups (A, C) or for ecological subregions (B, D). The complex sampling design was considered by incorporating sampling weights and bootstrap weights (N = 1291). ∗*P* < 0.05; ∗∗ *P* < 0.01; ∗∗∗ *P* < 0.001 indicate statistically significant geometric mean ratio (GMR) when comparing with the 16-19 y group +++ *P* < 0.001 indicates a statistically significant GMR compared with the other 2 regions.

Ergothioneine and selenoneine blood levels were positively associated with age in both sexes (Figure 3, panels A and C). Sex-combined GMR comparing the ≥60 y with the 16 to 19 y age groups were 1.75 (1.52, 2.02) and 2.78 (2.04, 3.69) for ergothioneine and selenoneine, respectively.

Blood selenoneine concentrations were greater in residents of communities along the Hudson Strait compared with the other 2 ecological subregions (Figure 3, panels B and D). Sex-combined GMR comparing Hudson Strait residents with Hudson Bay residents and Ungava Bay residents were 2.70 (2.22, 3.28) and 2.38 (1.97, 2.86), respectively. In contrast, ergothioneine blood concentrations were similar across ecological subregions.

Concentrations of all analytes measured in the blood of Nunavimmiut were moderately to strongly intercorrelated (Table 3). The strongest correlation coefficients were noted between parent compounds and their S/Se-methylated metabolites (Pearson r ≥0.90). Accordingly, we are only presenting results for ergothioneine and selenoneine in the following statistical analyses.

**TABLE 3:** Intercorrelations between blood concentrations of ergothioneine, selenoneine, and methylated metabolites in Nunavimmiut aged ≥16 y, *Qanuilirpitaa*? 2017 Health Survey^1^.

Blood metabolite	Ergothioneine	S-methyl-ergothioneine	Selenoneine	Se-methyl-selenoneine
Ergothioneine	—	—	—	—
S-methyl-ergothioneine	0.90	—	—	—
Selenoneine	0.69	0.58	—	—
Se-methyl-selenoneine	0.65	0.65	0.94	—

1 Pearson correlation coefficient on log-transformed concentrations (*N* = 1291). All coefficients are statistically significant (*P* < 0.001).

### Associations between blood metabolites and dietary habits

**FIGURE 4:**
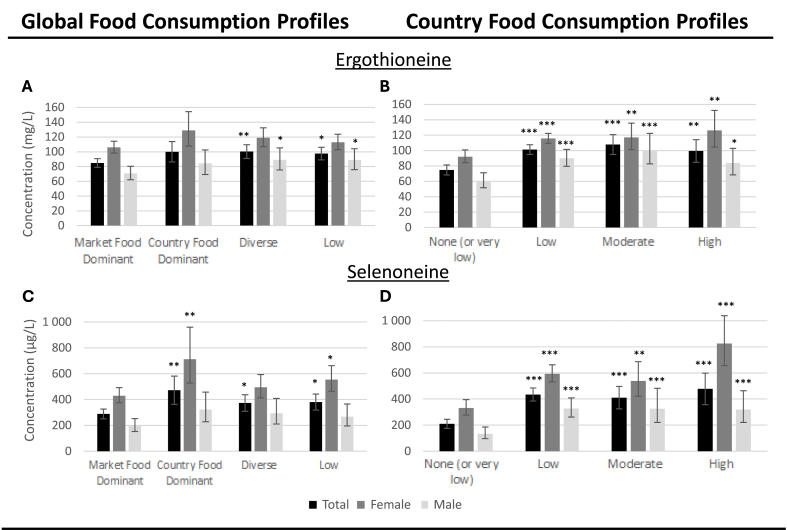
Adjusted geometric mean blood concentrations of ergothioneine and selenoneine (+/- 95% confidence interval) according to food consumption profiles in Nunavimmiut aged 16 and over, *Qanuilirpitaa*? 2017 Health Survey. Sex-specific models were adjusted for the current smoking status (daily, occasionally, not at all) and the following continuous variables: age, waist circumference, and blood hemoglobin concentration. The total model (males and females combined) was adjusted for sex in addition to the variables mentioned above. Statistical significance was tested using *t* values for the difference of means obtained from adjusted linear regression models. The complex sampling design was considered by incorporating sampling weights and bootstrap weights (N = 1121). ∗ *P* < 0.05; ∗∗ *P* < 0.01; ∗∗∗ *P* < 0.001 indicate statistically significant geometric mean ratio when comparing with the market food dominant category (global food consumption profiles; panels A and C) or the none (or very low) category (country food consumption profiles; panels B and D).

GM concentrations of selenoneine were higher among Nunavimmiut who had a country food dominant dietary profile than in those exhibiting a market food dominant profile (GMR = 1.68 [1.14, 2.29]), total - males and females combined; Figure 4, panels A and C). Ergothioneine concentrations were only marginally higher in the country food dominant dietary profile compared with the market food dominant profile (GMR = 1.18 [0.99, 1.39]). Mean concentrations of ergothioneine and selenoneine were also higher in the diverse consumption and low consumption profiles compared with the market food dominant profile, but GMR were smaller than those noted comparing country food dominant and market food dominant profiles. Similar trends were noted in males and females.

Mean ergothioneine and selenoneine concentrations generally increased with profiles of country food consumption, with the lowest mean concentrations observed in the none (or very low) consumption profile and the highest in the high consumption profile, but this trend was most apparent among females (Figure 4, panels B and D). A GMR value of 1.38 (1.14, 1.68) was noted for ergothioneine comparing females in the high with those of the none (or very low) country food profile, whereas a larger GMR of 2.63 (1.98, 3.47) was noted for selenoneine. In males, similar GMRs were noted, comparing either the low, medium, or high consumption profiles with the none (or very low) country food consumption profile.

Covariate-adjusted univariate models revealed that most country food items considered as potential sources of ergothioneine were positively associated with blood ergothioneine concentrations, whereas negative associations were mostly observed with potential market food sources (Supplemental Table 4). Most potential country food sources of selenoneine were also positively associated with blood selenoneine concentrations (Supplemental Table 5). Because univariate associations were similar in females and males, only global (sex-aggregated) multivariate models are shown in Table 4. Positive associations were noted between blood ergothioneine concentrations and consumption frequencies of Arctic char (GMR = 1.07), other fish (1.05), dried beluga meat (1.03), caribou meat consumed fresh, cooked, or frozen (1.06), and chicken eggs (1.04). Surprisingly, dried caribou meat consumption was negatively associated with blood ergothioneine levels (0.96). Consumption frequencies of Arctic char (1.07), dried beluga meat (1.09), beluga mattaaq (1.15) and seaweed (1.11) were positively related to blood selenoneine concentrations. Except for the positive association mentioned above between chicken egg consumption and blood ergothioneine levels, consumption frequencies of potential market food sources were negatively associated with blood antioxidant concentrations.

**TABLE 4:** Associations between blood concentrations of ergothioneine and selenoneine and consumption frequencies of selected foods among Nunavimmiut aged 16 and over, *Qanuilirpitaa*? 2017 Health Survey^1^.

Antioxidant		Food item		GMR		95% CI
Ergothioneine						
		Country food				
			Arctic char	1.07		1.04, 1.10
			Other fish	1.05		1.01, 1.10
			Dried beluga meat	1.03		1.01, 1.06
			Caribou meat (fresh, cooked, frozen)	1.06		1.03, 1.10
			Dried caribou meat	0.96		0.93, 0.99
						
		Market food				
			Chicken eggs	1.04		1.01, 1.06
			Chicken or turkey	0.96		0.93, 1.00
			Red and processed meat	0.95		0.91, 0.98
			Beans or lentils or chickpeas	0.97		0.93, 1.00
			Hot cereals (oatmeal)	0.97		0.94, 0.99
Selenoneine						
		Country food				
			Arctic char	1.07		1.02, 1.12
			Dried beluga meat	1.09		1.02, 1.15
			Beluga mattaaq	1.15		1.08, 1.22
			Seaweed	1.11		1.04, 1.18
		Market food				
			Canned fish (tuna, salmon or sardines)	0.93		0.87, 1.00

Multivariate linear regression models (*N* = 1121) include all food items and were adjusted for sex, the current smoking status (daily, occasionally, not at all), and the following continuous variables: age, waist circumference, and blood hemoglobin concentration. Statistical significance was assessed using the *t* value of model coefficients. The complex sampling design was accounted for with sampling weights and bootstrap weights. Univariate models are shown in Supplemental Tables 4 and 5 (Supplementary Material). GMR, geometric mean ratio. Associations with covariates are shown in Supplemental Table 6 (Supplementary Material).

GMR=geometric mean ratio.

1 Estimates are adjusted geometric mean ratio per doubling of monthly food consumption frequency.

Among covariates included in multivariate models, age and sex were associated with blood concentrations of both antioxidants, whereas blood hemoglobin and WC were only associated with blood ergothioneine concentrations (Supplemental Table 6). Similar models were obtained when using BMI categories instead of WC to adjust for obesity (data not shown).

## Discussion

We measured blood concentrations of ergothioneine and selenoneine, 2 closely related antioxidants with many potential health benefits, and their methylated metabolites among Nunavik youth and adults sampled during a large population survey conducted in 2017. Females, older people, and frequent country food consumers displayed the highest concentrations of these compounds. Although limited, comparisons with small groups of individuals elsewhere in the world suggest that Nunavimmiut exhibit among the highest concentrations of these dietary antioxidants, due to their frequent consumption of several marine and terrestrial country food species that are central to Inuit identity, culture, and food security.

### Comparisons with other populations

To our knowledge, this is the first report of blood ergothioneine concentrations in a representative population sample anywhere in the world. Cheah et al. [24] examined the variation of ergothioneine concentrations in whole blood of 135 nonrandomly selected elderly individuals in Singapore (Jurong District) in relation to age, using a sensitive and specific LC-MS/MS method. From age 60 to 90, the mean concentration decreased from ∼ 23 to 11 mg/L. In the same study, a similar age-related trend was noted for plasma ergothioneine concentrations, with mean values decreasing from 0.10 mg/L in individuals <65y to 0.06 mg/L in those >75y [24]. This research group also reported a mean ergothioneine concentration of 22.6 mg/L in the blood of 15 healthy male volunteers of Chinese ethnicity aged 21 to 35 y [10]. Using the same method as the one used in the present work, we reported a mean (geometric) concentration of 22.6 mg/L in 10 volunteers (non-Indigenous) from our laboratory, aged between 21 and 56 years [16]. Comparisons with these limited datasets suggest that blood ergothioneine levels in Nunavimmiut are, on average, ∼5 times higher than in other populations. Comparative data are even fewer for selenoneine. A mean concentration of 12.5 μg/L was measured in blood samples obtained from the 10 volunteers in our laboratory [16], a value ∼30-fold lower than the GM blood selenoneine concentration of 355 μg/L among Nunavimmiut in the *Qanuilirpitaa*? 2017 survey. Our group previously reported a GM concentration of selenoneine of 118 μg Se/L in RBCs of 885 Nunavimmiut aged 18 and over who participated in the *Qanuippitaa?* 2004 survey [15]. A geometric mean selenoneine concentration of 0.212 μg Se/g was measured in blood cells of a fish-eating population living on remote Japanese islands (*N* = 167; mean age = 63.2 years) [25]. It is our hope that the analytical method developed for this study and the availability of selenoneine standards [16] will allow documenting blood concentrations of these unique antioxidant compounds in other Indigenous and non-Indigenous populations in the coming years.

Comparisons of total blood selenium concentrations observed in the present survey to those documented in population surveys conducted during the same period in Canada and the United States suggest that selenoneine concentrations among Nunavimmiut are substantially higher than in general Canadian and American populations. Median, 75^th,^ and 95^th^ percentile values of blood selenium concentrations are respectively 170, 190, and 220 μg/L for individuals aged 16 to 79 who participated in the Canadian Health Measures Survey Cycle 6 in 2018-2019 [26]. Corresponding blood concentration values among Nunavimmiut (Supplemental Table 3) are respectively 1.6-, 2.2-, and 3.6-fold higher than those of the general Canadian population. In the United States, Bai et al. [27] reported median, 75^th^ and 97.5^th^ percentile values of 184, 201, and 241 μg/L for total blood selenium concentrations among 6205 individuals aged 18 to 79 years who participated in the NHANES 2017–2020. Corresponding values in Nunavimmiut (Supplemental Table 3) are respectively 1.5-, 2.0-, and 3.8-fold greater than those of the general American population. More definitive conclusions about the magnitude of differences in blood selenoneine concentrations across populations will require specific quantification of this selenium species in future population surveys.

### Concentrations by sex, age, and ecological subregions

Females displayed nearly 2-fold greater blood concentrations of selenoneine and its methylated metabolite compared with males. This difference is in line with our previous results indicating a similarly greater selenoneine content in RBCs of females compared with males who participated in the *Qanuippitaa?* Nunavik Health Survey in 2004 [15]. A possible explanation advanced by Little et al. [28] is the consumption of the selenoneine-rich mattaaq from beluga tail exclusively by females.

Older age groups exhibited higher concentrations of all analyzed compounds, which could indicate either their accumulation with age or a greater intake of country foods that are rich in ergothioneine and selenoneine in older Nunavimmiut. Little is known regarding the pharmacokinetics of these compounds. The high expression concentration of the ETT in the kidneys [9] suggests an efficient reabsorption of both compounds. Methylation is the only known biotransformation pathway of ergothioneine [10] and selenoneine [12, 29], which may be a prerequisite for their urinary excretion. Therefore, it is not clear at the present time if these compounds accumulate with age in the body. In contrast, data from this health survey and the previous one conducted in the same population in 2004 indicate that the consumption of most country foods is more frequent in older people [14,17,18]. Age was positively associated with blood ergothioneine and selenoneine concentrations in covariate-adjusted multivariate models (Supplemental Table 6), suggesting either age-related differences in dietary sources unaccounted for in the models or accumulation of these compounds with age.

The higher blood selenoneine concentrations noted in Nunavimmiut living in communities along the Hudson Strait compared with Hudson Bay and Ungava Bay residents likely reflects differences in beluga mattaaq consumption frequencies among ecological subregions: Hudson Strait, GM = 2 times/mo compared with 1 time/mo in Hudson Bay and Ungava Bay communities [14]. Conversely, no difference was noted among ecological subregions for blood concentrations of ergothioneine, possibly because caribou meat consumption frequency is more evenly distributed across Nunavik subregions (Hudson Strait, GM = 2.54 times/wk compared with Hudson Bay, 2.36 times/wk and Ungava Bay, 1.78 times/wk) [14]. Moreover, ergothioneine is found in both marine and terrestrial wild foods (Supplemental Table 1), whereas selenoneine presence appears limited to foods of marine origin [5].

### Associations between blood antioxidant levels and dietary habits

Associations between blood antioxidants concentrations and food consumption profiles suggest that country foods regularly consumed by Nunavimmiut are responsible for the relatively high blood concentrations of both compounds in this population (Figure 4). The higher blood selenoneine concentrations noted in Nunavimmiut exhibiting a country food dominant profile is likely due to the greater consumption frequency of marine foods by these individuals compared with those in the market food profile [18]. Blood selenoneine levels were also greater in the diverse and low consumption profiles, which comprise some beluga mattaaq consumption, compared with the market food dominant profile. Lower blood ergothioneine concentrations were noted in individuals belonging to the market food dominant profile, compared with all other global food consumption profiles, again suggesting a greater variety of country food sources for this antioxidant. As for country food consumption profiles, Nunavimmiut in either the low, moderate, or high-country food consumption profile exhibited greater blood concentrations of both antioxidants and their metabolites than those in the none (or very low) consumption profile. In contrast to ergothioneine, for which there was little evidence of a graded response, an increasing trend with profiles of country food consumption (from low to moderate to high) was noted for blood selenoneine concentrations.

Covariate-adjusted multivariate models that included consumption frequencies of all possible food sources indicated that in addition to the selenoneine-rich beluga mattaaq, other marine country foods with relatively low selenoneine content (i.e., Arctic char, dried beluga meat, seaweed) seemed to contribute to blood selenoneine levels (Table 4). In contrast, wild foods from the sea (i.e., Arctic char, other fish, dried beluga meat) and the land (i.e., caribou meat) were related to blood ergothioneine levels. FFQ is a tool designed to assess diet in general population studies and may not be optimal to identify specific food compounds [30]. In this context, the negative associations noted with the consumption of most market foods in multivariate models again point out the importance of country foods as dietary sources of ergothioneine and selenoneine: the greater the consumption of market foods, the lower the consumption of antioxidant-rich country foods, and in turn the lower the concentrations of both antioxidants in the blood of Nunavimmiut.

### Strengths/limitations of our study

Our study has some limitations. First, the participation rate to the *Qanuilirpitaa?* 2017 survey was relatively low (36.5%), which increases the risk of selection bias. However, discussions with Inuit partners during cointerpretation sessions indicate that the survey succeeded in capturing the variety of dietary habits in this population [18]. Second, the FFQ referred to a 3-mo period prior to the survey, including late spring and summer. Because both ergothioneine and selenoneine accumulate in RBCs, which have a lifespan of ∼120 d [31], blood concentrations should mostly reflect the period of reference of the dietary questionnaire. However, country food being highly seasonal in Nunavik [32], concentrations of ergothioneine/selenoneine and associations with diet may differ depending on the time of year. Additionally, we only have limited knowledge of the content of ergothioneine and selenoneine in country foods, and important additional dietary sources may be discovered in the near future. The strengths of our study are *1*) the large population sample covering all communities of Nunavik and *2*) the specific, precise, and fully validated analytical method used for the determination of target analytes in whole blood [16]. Our results may be applicable to other Inuit populations living elsewhere in the Arctic (other regions of Inuit Nunangat, Greenland) and to Indigenous populations throughout the world that rely on wild food for subsistence.

Our results indicate that Nunavimmiut displayed in 2017 relatively high concentrations of both ergothioneine and selenoneine, 2 bioactive food components abundant in popular country foods in Nunavik. The possible benefits of these antioxidants for the health of Nunavimmiut are currently being investigated.

## Author contribution

The authors’ responsibilities were as follows – PA, M Lemire, M Little: conceived the research; PA, M Lemire, PD, A Achouba, MY, A Aker, ABB, NO: acquired data; PA, M Lemire, M Little, A Aker, AB: analyzed the database; all authors: contributed to data interpretation; PA, M Lemire, A Achouba, M Little: wrote the manuscript; and all authors: reviewed and approved the final manuscript.

## Data availability

Data described in the manuscript, code book, and analytic code will not be made available because of participant privacy and confidentiality concerns. Further information, including the procedures to obtain and access data from the *Qanuilirpitaa?* 2017 Health Survey can be obtained by contacting the Nunavik Regional Board of Health and Social Services (email: nunavikhealthsurvey@ssss.gouv.qc.ca).

## Funding

This research was supported by ArcticNet – a Network of Centres of Excellence of Canada – grant number P11 and Crown-Indigenous Relations and Northern Affairs Canada Northern Contaminants Program Human Health grant number H-04. M Little is supported by a Michael Smith Health Research BC Scholar Award. M Lemire is a member of Quebec Océan and the titular of the Littoral Research Chair – the Sentinel North Partnership Research Chair in Ecosystem Approaches to Health (2019-2024) – which is funded by Sentinel North and the Northern Contaminants Program of Crown-Indigenous Relations and Northern Affairs Canada. M Lemire is currently supported by a Senior Research Scholar Award from the Quebec Health Research Fund.

## Conflict of interest

A Achouba and PA have patent TOTAL SYNTHESES OF SELENONEINE, ISO-SELENONEINE, AND ISOMERS #PCT/CA2023/050197 pending to Université Laval. All other authors report no conflicts of interest.
