# Supplementary material for: Wild foods as drivers of blood ergothioneine and selenoneine concentrations among Inuit living in Nunavik: results from the cross-sectional Qanuilirpitaa? 2017 survey
Source: Am J Clin Nutr. 2025 Jul 4;122(2):582–92. doi: 10.1016/j.ajcnut.2025.05.009 (PMC12405779; doi:10.1016/j.ajcnut.2025.05.009)
Supplement: Multimedia component 1 [file mmc1.docx]

**Wild foods as drivers of blood ergothioneine and selenoneine concentrations among Inuit living in Nunavik: results from the cross-sectional *Qanuilirpitaa?* 2017 survey**

Pierre Ayotte^1,2,3*^, Mélanie Lemire^1,2,4^, Pierre Dumas^3^, Adel Achouba^2^, Marcos Yedjenou^2^, Ariane B. Barrette^5^, Nathalie Ouellet^2^, Matthew Little^6^, Amira Aker^2**^

**Supplementary Material**

**Supplemental Tables 1-6**

**Supplemental Table 1:** Ergothioneine and selenoneine content of selected country foods^1^

**Species Part N Sampling Location/Year Selenoneine (mg/kg ww) Ergothioneine (mg/kg ww) References**

Low Medium High Low Medium High

0.1-1 1-10 10-100 0.1-1 1-10 10-100

Beluga Muscle 14 Quaqtaq (2018-2019) X X Barrette et al. ^2^

Beluga Skin 14 Quaqtaq (2018-2019) X X Achouba et al. 2019^3^; Barrette et al.^2^

Seal Muscle 20 Quaqtaq (2017) X Cinq-Mars et al. 2022^3^; Barrette et al.^1^

Seal Liver 20 Quaqtaq (2017) X Cinq-Mars et al. 2022^3^; Barrette et al.^4^

Caribou Muscle 61 Northern Quebec (2011) <LOD X Ayotte et al. (unpublished)

Arctic char Muscle 30 Western Canadian Arctic (2021) X X Gamberg et al. (unpublished)

Whitefish Muscle 10 Western Canadian Arctic (2021) X X Gamberg et al. (unpublished)

Lake trout Muscle 14 Western Canadian Arctic (2021) X X Gamberg et al. (unpublished)

Sculpin Muscle 4 Hudson Strait (2021) X X Barrette et al. (unpublished)

Mussel Whole 44 Hudson Strait (2022–2023) X X Barrette et al. (unpublished)

Scallop Whole 5 Quaqtaq (2021) X X Barrette et al. (unpublished)

Urchin Whole 9 Quaqtaq (2021) X X Barrette et al. (unpublished)

Seaweed Blade 6 Hudson Strait/Baffin Bay (2021) X X Barrette et al. (unpublished)

^1^ Country foods consumed by at least 25% of Nunavimmiut over the last 3 months prior to the Qanuiliprpitaa?2017 survey.

^2^ Article in preparation.

^3^ Concentrations in Achouba et al (2019) and Cinq-Mars et al (2002) were determined using our previous LC-ICP-MS/MS method described in Achouba et al. (2019). Most recent concentrations (unpublished) were measured using a modification of the ID-LC-MS/MS described in Achouba et al. (2023).

^4^ Article in progress.

**Supplemental Table 2:** Correspondence between consumption categories from the food frequency questionnaire (FFQ) and monthly consumption frequency

FFQ category Range of consumption frequency Times per month

1 None or <1/month 0.5

2 1-3/month 2

3 1/week 4

4 2-6/week 16

5 1/day 30

6 2-3/day 75

7 >=4/day 120^1^

^1^ Maximum value set at the 99^th^ percentile for summation variables (red and processed meat; chicken or turkey).

**Supplemental Table 3:** Blood concentrations of selenoneine, total selenium, and selenoneine to total selenium molar ratio in Nunavimmiut aged 16 and over, *Qanuilirpitaa*? 2017 Health Survey

**Variable GM^1^ Median 75^th^ perc. 95^th^ perc. 97.5^th^ perc.**

**(95%CI) (95%CI) (95%CI) (95%CI) (95%CI)**

Selenoneine (µg/L) 355 424 959 2229 2754

(328-385) (385-462) (879-1039) (2069-2388) (2389-3119)

Selenoneine (µmol/L) 1.29 1.54 3.48 8.10 10.0

(1.19-1.40) (1.40-1.68) (3.19-3.78) (7.52-8.68) (8.68-11.3)

Total selenium (µg/L)^2^ 296 272 413 785 915

(288-305) (261-282) (389-438) (733-836) (827-1005)

Total selenium (µmol/L) 3.75 3.44 5.23 9.94 11.6

(3.65-3.86) (3.31-3.57) (4.93-5.55) (9.28-10.6) (10.5-12.7)

Selenoneine/total selenium 0.34 0.45 0.65 0.82 0.89

molar ratio (0.32-0.36) (0.42-0.47) (0.63-0.67) (0.84-0.86) (0.86-0.91)

^1^ Geometric mean N = 1291.

^2^ Analytical methods for total selenium detailed in Lavoie et al. (2024).

**Supplemental Table 4:** Adjusted univariate associations^1^ between blood concentrations of ergothioneine and consumption frequencies of selected foods among Nunavimmiut aged 16 and over, *Qanuilirpitaa*? 2017 Health Survey

Total Females Males

Food item **GMR^2^ 95%CI GMR 95%CI GMR 95%CI**

Country food

Arctic char 1.08 1.05-1.11 1.07 1.04-1.10 1.09 1.04-1.14

Other fish 1.06 1.01-1.11 1.06 1.02-1.10 1.06 0.98-1.13

Dried fish 1.05 1.02-1.09 1.04 1.01-1.08 1.06 1.01-1.12

Lake trout 1.02 0.99-1.05 0.98 0.94-1.01 1.06 1.01-1.12

Beluga meat (fresh, cooked, or frozen) 1.00 0.96-1.03 0.99 0.96-1.03 1.00 0.95-1.05

Dried beluga meat 1.03 1.00-1.06 1.04 1.01-1.07 1.03 0.99-1.08

Beluga mattaaq 1.05 1.01-1.08 1.04 1.01-1.07 1.05 1.00-1.11

Seal meat 1.03 0.99-1.06 1.02 0.98-1.02 1.03 0.98-1.08

Seal liver 1.03 0.99-1.07 1.01 0.97-1.05 1.04 0.98-1.10

Caribou meat (fresh, cooked, or frozen) 1.06 1.04-1.09 1.06 1.02-1.09 1.07 1.03-1.12

Dried caribou meat 1.03 1.00-1.06 1.02 0.99-1.06 1.03 0.99-1.08

Ptarmigan or partridge 1.01 0.98-1.05 1.00 0.97-1.04 1.02 0.96-1.08

Snow or Canada goose 1.04 1.01-1.06 1.02 0.99-1.06 1.05 1.00-1.10

Mollusks or urchins 1.03 1.00-1.07 1.02 0.99-1.05 1.04 0.98-1.10

Seaweed 1.03 1.00-1.07 1.03 1.00-1.06 1.04 0.98-1.11

Market food

Chicken eggs 1.02 1.00-1.04 1.01 0.99-1.03 1.03 0.99-1.07

Chicken or turkey 0.96 0.93-1.00 0.96 0.94-1.00 0.97 0.91-1.02

Red and processed meat 0.96 0.92-1.00 0.98 0.94-1.01 0.95 0.89-1.01

Canned fish 0.97 0.93-1.01 1.00 0.96-1.05 0.95 0.89-1.00

Beans or lentils or chickpeas 0.97 0.93-1.00 0.98 0.95-1.01 0.96 0.91-1.01

Nuts or peanuts or sunflower seeds 0.98 0.95-1.01 0.99 0.96-1.02 0.97 0.92-1.02

Other vegetables or mushrooms 1.01 0.98-1.03 1.01 0.98-1.03 1.01 0.96-1.05

Hot cereals (oatmeal) 0.97 0.95-1.00 1.00 0.97-1.02 0.95 0.91-0.99

^1^ Models were adjusted for sex, the current smoking status (daily. occasionally. not at all), and the following continuous variables: age. waist circumference, and blood hemoglobin concentration (N = 1121).
^2^ GMR: adjusted geometric mean ratio per doubling of monthly food consumption frequency.

**Supplemental Table 5:** Adjusted univariate associations^1^ between blood concentrations of selenoneine and consumption frequencies of selected foods among Nunavimmiut aged 16 and over, *Qanuilirpitaa*? 2017 Health Survey

Total Females Males

Food item **GMR^2^ 95%CI GMR 95%CI GMR 95%CI**

Country food

Arctic char 1.13 1.07-1.19 1.08 1.03-1.14 1.17 1.08-1.27

Other fish 1.07 0.99-1.15 1.05 0.98-1.13 1.09 0.97-1.22

Dried fish 1.13 1.07-1.20 1.09 1.04-1.15 1.16 1.05-1.28

Lake trout 1.01 0.96-1.07 0.97 0.91-1.03 1.05 0.96-1.16

Beluga meat (fresh, cooked, or frozen) 1.10 1.04-1.17 1.10 1.04-1.17 1.10 1.00-1.22

Dried beluga meat 1.17 1.10-1.24 1.18 1.12-1.24 1.17 1.06-1.29

Beluga mattaaq 1.23 1.16-1.31 1.22 1.14-1.31 1.24 1.13-1.37

Seal meat 1.08 1.01-1.16 1.08 1.00-1.15 1.08 0.97-1.20

Seal liver 1.09 1.01-1.18 1.10 1.02-119 1.08 0.97-1.21

Ptarmigan or partridge 1.03 0.96-1.10 1.01 0.94-1.08 1.04 0.92-1.16

Snow or Canada goose 1.04 0.98-1.10 1.01 0.95-1.07 1.07 0.98-1.17

Mollusks or urchins 1.13 1.06-1.20 1.14 1.08-1.21 1.12 1.01-1.24

Seaweed 1.19 1.11-1.27 1.20 1.13-1.27 1.17 1.03-1.33

Market food

Canned fish 0.96 0.90-1.03 0.99 0.92-1.06 0.95 0.85-1.05

^1^ Models were adjusted for sex, the current smoking status (daily. occasionally. not at all), and the following continuous variables: age. waist circumference, and blood hemoglobin concentration (N = 1121).
^2^ GMR: adjusted geometric mean ratio per doubling of monthly food consumption frequency.

**Supplemental Table 6** Associations between blood concentrations of ergothioneine and selenoneine and consumption frequencies of selected foods among Nunavimmiut aged 16 and over, *Qanuilirpitaa*? 2017 Health Survey ^1^

Antioxidant Food item or covariate GMR 95% CI

Ergothioneine

Country food

Arctic char 1.07 1.04, 1.10

Other fish 1.05 1.01, 1.10

Dried beluga meat 1.03 1.01, 1.06

Caribou meat (fresh, cooked, frozen) 1.06 1.03, 1.10

Dried caribou meat 0.96 0.93-0.99

Market food

Chicken eggs 1.04 1.01, 1.06

Chicken or turkey 0.96 0.93, 1.00

Red and processed meat 0.95 0.91, 0.98

Beans or lentils or chickpeas 0.97 0.93, 1.00

Hot cereals (oatmeal) 0.97 0.94, 0.99

Covariate

Sex (ref. male) 1.39 1.25, 1.54

Age 1.01 1.00, 1.01

Blood hemoglobin 1.00 1.00, 1.01

Waist circumference 1.01 1.00, 1.01

Selenoneine

Country food

Arctic char 1.07 1.02, 1.12

Dried beluga meat 1.09 1.02, 1.15

Beluga mattaaq 1.15 1.08, 1.22

Seaweed 1.11 1.04, 1.18

Market food

Canned fish (tuna, salmon or sardines) 0.93 0.87, 1.00

Covariate

Sex (ref. male) 2.07 1.67, 2.57

Age 1.02 1.01, 1.03

^1^ Estimates are adjusted geometric mean ratio per doubling of monthly food consumption frequency.
Multivariate linear regression models (N = 1121) include all food items and were adjusted for sex, the current smoking status (daily, occasionally, not at all), and the following continuous variables: age, waist circumference, and blood hemoglobin concentration. Statistical significance was assessed using the *t* value of model coefficients. The complex sampling design was accounted for with sampling weights and bootstrap weights. Univariate models are shown in Supplemental Tables 4 and 5 (Supplementary Material). GMR, geometric mean ratio.

**References**

Achouba A, Dumas P, Ouellet N, Little M, Lemire M, Ayotte P. Selenoneine is a major selenium species in beluga skin and red blood cells of Inuit from Nunavik. Chemosphere. 2019;229:549-558.

Cinq-Mars G. Les aliments de la mer au Nunavik : mieux comprendre les variations des concentrations d'éléments essentiels et de méthylmercure chez les phoques annelés, les bélugas et les morses [Master’s thesis, Université Laval]. 2022. Corpus UL <https://corpus.ulaval.ca/server/api/core/bitstreams/24490385-be79-464c-a61e-413f0c095683/content>.

Lavoie A, Lemire M, Lévesque B, Ayotte P. Determinants of iron deficiency and anemia among Nunavimmiut: results from the Qanuilirpitaa? 2017 Nunavik Health Survey. Can J Public Health. 2024;115(Suppl 1):152-167.
